# 2050 Identifying the role and immunobiological mechanisms of Fli-1 mediated pathogenicity in graft Versus host disease

**DOI:** 10.1017/cts.2018.83

**Published:** 2018-11-21

**Authors:** Steven Schutt, Yongxia Wu, Anusara Daenthanasanmak, David Bastian, Carole Wilson, Lynn Schnapp, Xian Zhang, Xue-Zhong Yu, Hung Nguyen, Mohammed Hanief Sofi, Supinya Iamsuwat

**Affiliations:** 1 Medical University of South Carolina

## Abstract

OBJECTIVES/SPECIFIC AIMS: Allogeneic hematopoietic stem cell transplantation (allo-HCT) is a curative procedure for hematological malignancies. Chronic graft Versus host disease (cGVHD) is a lethal complication that often develops after allo-HCT. Fli-1 is an aberrantly expressed protein in cancers including erythroleukemia and melanoma, while being implicated in pathogenesis of systemic lupus in mice and humans, a disease with marked similarity to cGVHD. METHODS/STUDY POPULATION: cGVHD was induced using hematopoietic cells from conditional knock-out mice deficient for the fli-1 gene specifically on T cells and progression of cGVHD in murine allo-HCT recipients was monitored using a clinical scoring system, and changes in activation status of hematopoietic cell populations were quantified using flow cytometry. RESULTS/ANTICIPATED RESULTS: Recipients transplanted with fli-1 deficient T cells exhibited reduced cGVHD clinical scores compared with littermate wild-type controls. Donor-grafts containing fli-1 deficient T cells were associated with restrained T-cell responses including reduced Interferon-y cytokine production, PD-1 expression, and differentiation into follicular helper T cells. fli-1 T-cell deficient donor-grafts also improved donor B-cell reconstitution and reduced plasma cells in allo-HCT recipients relative to littermate wild-type control donor-graft recipients. DISCUSSION/SIGNIFICANCE OF IMPACT: Thus, inhibiting Fli-1 represents a promising therapeutic strategy for the goal of preventing cGVHD after allo-HCT while also directly targeting cancers which aberrantly express Fli-1.

